# Staying Ahead of the Epidemiologic Curve: Evaluation of the British Columbia Asthma Prediction System (BCAPS) During the Unprecedented 2018 Wildfire Season

**DOI:** 10.3389/fpubh.2021.499309

**Published:** 2021-03-12

**Authors:** Sarah B. Henderson, Kathryn T. Morrison, Kathleen E. McLean, Yue Ding, Jiayun Yao, Gavin Shaddick, David L. Buckeridge

**Affiliations:** ^1^Environmental Health Services, British Columbia Centre for Disease Control (BCCDC), Vancouver, BC, Canada; ^2^Department of Epidemiology and Biostatistics, McGill University, Montreal, QC, Canada; ^3^Department of Mathematical Sciences, University of Exeter, Exeter, United Kingdom

**Keywords:** wildfire smoke, public health, surveillance, forecasting, data integration

## Abstract

**Background:** The modular British Columbia Asthma Prediction System (BCAPS) is designed to reduce information burden during wildfire smoke events by automatically gathering, integrating, generating, and visualizing data for public health users. The BCAPS framework comprises five flexible and geographically scalable modules: (1) historic data on fine particulate matter (PM_2.5_) concentrations; (2) historic data on relevant health indicator counts; (3) PM_2.5_ forecasts for the upcoming days; (4) a health forecasting model that uses the relationship between (1) and (2) to predict the impacts of (3); and (5) a reporting mechanism.

**Methods:** The 2018 wildfire season was the most extreme in British Columbia history. Every morning BCAPS generated forecasts of salbutamol sulfate (e.g., Ventolin) inhaler dispensations for the upcoming days in 16 Health Service Delivery Areas (HSDAs) using random forest machine learning. These forecasts were compared with observations over a 63-day study period using different methods including the index of agreement (IOA), which ranges from 0 (no agreement) to 1 (perfect agreement). Some observations were compared with the same period in the milder wildfire season of 2016 for context.

**Results:** The mean province-wide population-weighted PM_2.5_ concentration over the study period was 22.0 μg/m^3^, compared with 4.2 μg/m^3^ during the milder wildfire season of 2016. The PM_2.5_ forecasts underpredicted the severe smoke impacts, but the IOA was relatively strong with a population-weighted average of 0.85, ranging from 0.65 to 0.95 among the HSDAs. Inhaler dispensations increased by 30% over 2016 values. Forecasted dispensations were within 20% of the observed value in 71% of cases, and the IOA was strong with a population-weighted average of 0.95, ranging from 0.92 to 0.98. All measures of agreement were correlated with HSDA population, where BCAPS performance was better in the larger populations with more moderate smoke impacts. The accuracy of the health forecasts was partially dependent on the accuracy of the PM_2.5_ forecasts, but they were robust to over- and underpredictions of PM_2.5_ exposure.

**Conclusions:** Daily reports from the BCAPS framework provided timely and reasonable insight into the population health impacts of predicted smoke exposures, though more work is necessary to improve the PM_2.5_ and health indicator forecasts.

## Introduction

Transient wildfire smoke causes episodes of the worst air quality that many populations will ever experience. A growing body of literature indicates that short-term smoke exposures are associated with a wide range of acute health impacts, from increased respiratory symptoms through to increased risk of premature mortality (1, 2). Timely intervention may help to prepare populations before smoke arrives, but it is challenging to evaluate potential risks over the upcoming days because the necessary information is disparately available from different sources (3, 4). Useful data include: observed air quality measurements from regulatory monitoring sites; observed smoke impacts from remote sensing platforms; predicted air quality impacts from smoke forecasting systems; observed counts of sensitive health indicators from administrative records; and established exposure-response relationships for different populations, given their underlying vulnerabilities.

**Figure 1:**
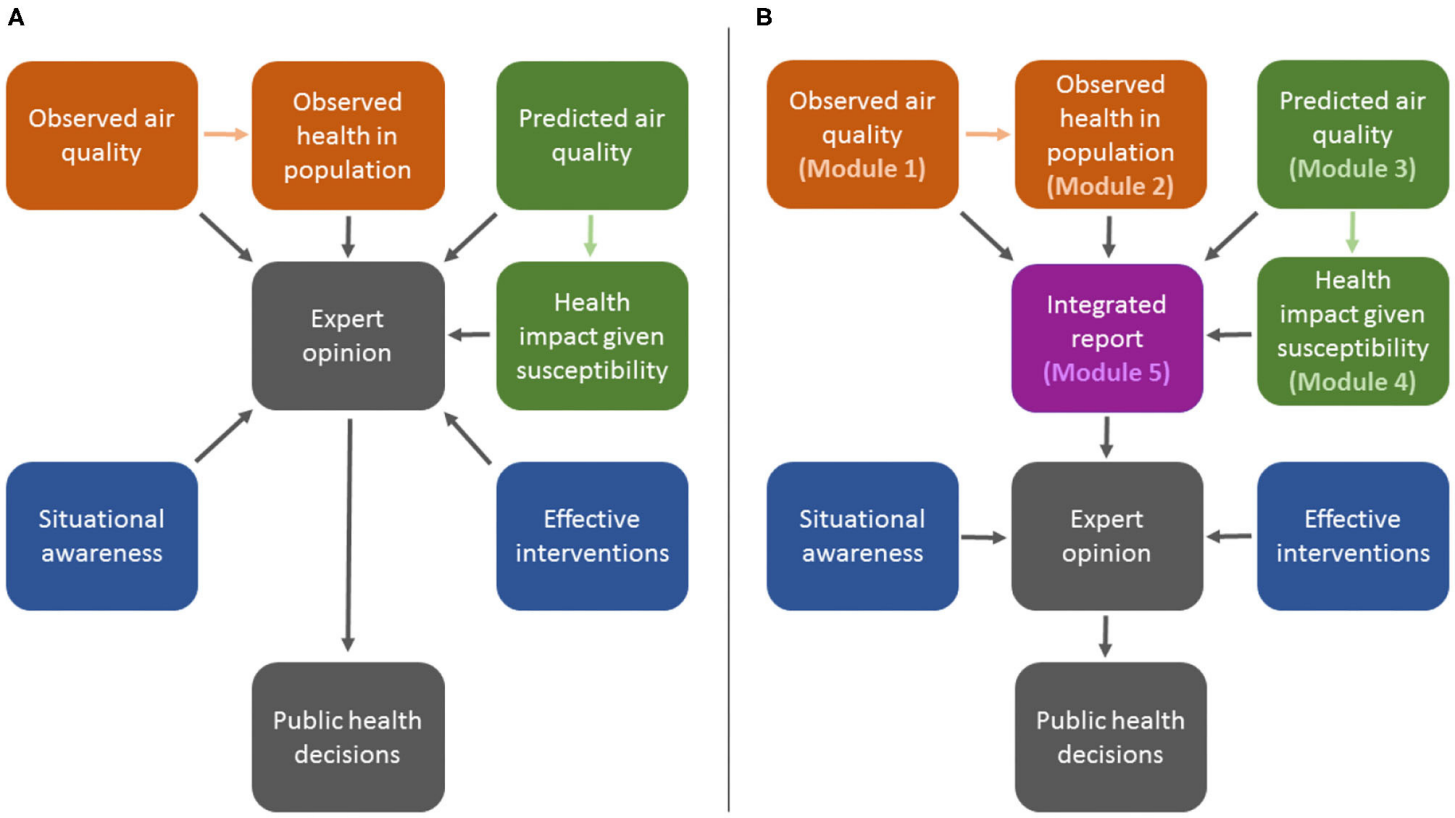
Illustration of the process for public health decision-making during wildfire smoke events without the British Columbia Asthma Prediction System (BCAPS) framework **(A)** and with the BCAPS framework described here **(B)**.

Many medical officers of health do not have the necessary time, resources, or technical expertise to manually gather, integrate, and interpret these data in real time to inform public health practice. As such, the public health response to smoke events could be improved by automated surveillance systems that collate the relevant data and provide useful information about the health impacts of observed and predicted smoke exposures for specific populations. The British Columbia Asthma Prediction System (BCAPS) is a framework that forecasts and visualizes the population health impacts of wildfire smoke over the next 24-hour (i.e., today, denoted *day*_0_) and 48-hour (i.e., tomorrow, denoted *day*_+1_) periods using five modules (Figure 1). *Module 1* holds historic daily records of fine particulate matter (PM_2.5_) concentrations. The complementary *Module 2* holds historic daily counts of a respiratory health indicator for the target population. On the forecast side, *Module 3* holds predicted PM_2.5_ concentrations for the upcoming days, derived from smoke forecasting models. *Module 4* establishes the statistical relationship between *Module 1* and *Module 2* to forecast the health impacts of *Module 3* over the coming days for the target population. Finally, *Module 5* visualizes information from each of the other modules in a daily report designed for easy interpretation by public health authorities.

Each of these modules is completely flexible, such that different data, modeling approaches, and visualizations can be applied depending on data availability, technical considerations, and user needs. Furthermore, the entire system is scalable to different geographies. The BCAPS framework was developed for use in the Canadian province of British Columbia, and has been operational since the extreme wildfire season of 2017. However, the framework could be adapted to any context where the necessary data inputs are available for public health surveillance. To provide more information on the utility of BCAPS for other jurisdictions, we have assessed its performance during the record-setting wildfire season of 2018 in British Columbia, during which most parts of the province were affected by smoke pollution ranging from moderate to severe. In practice, BCAPS reports were distributed to approximately 30 public health users across British Columbia at 09:00 each morning during the wildfire season. To provide the fairest possible assessment of BCAPS utility, the methods described and data analyzed here reflect exactly the information received by BCAPS users in 2018, though the system has since been improved and updated.

## Methods

### Study Area and Period

**Figure 2:**
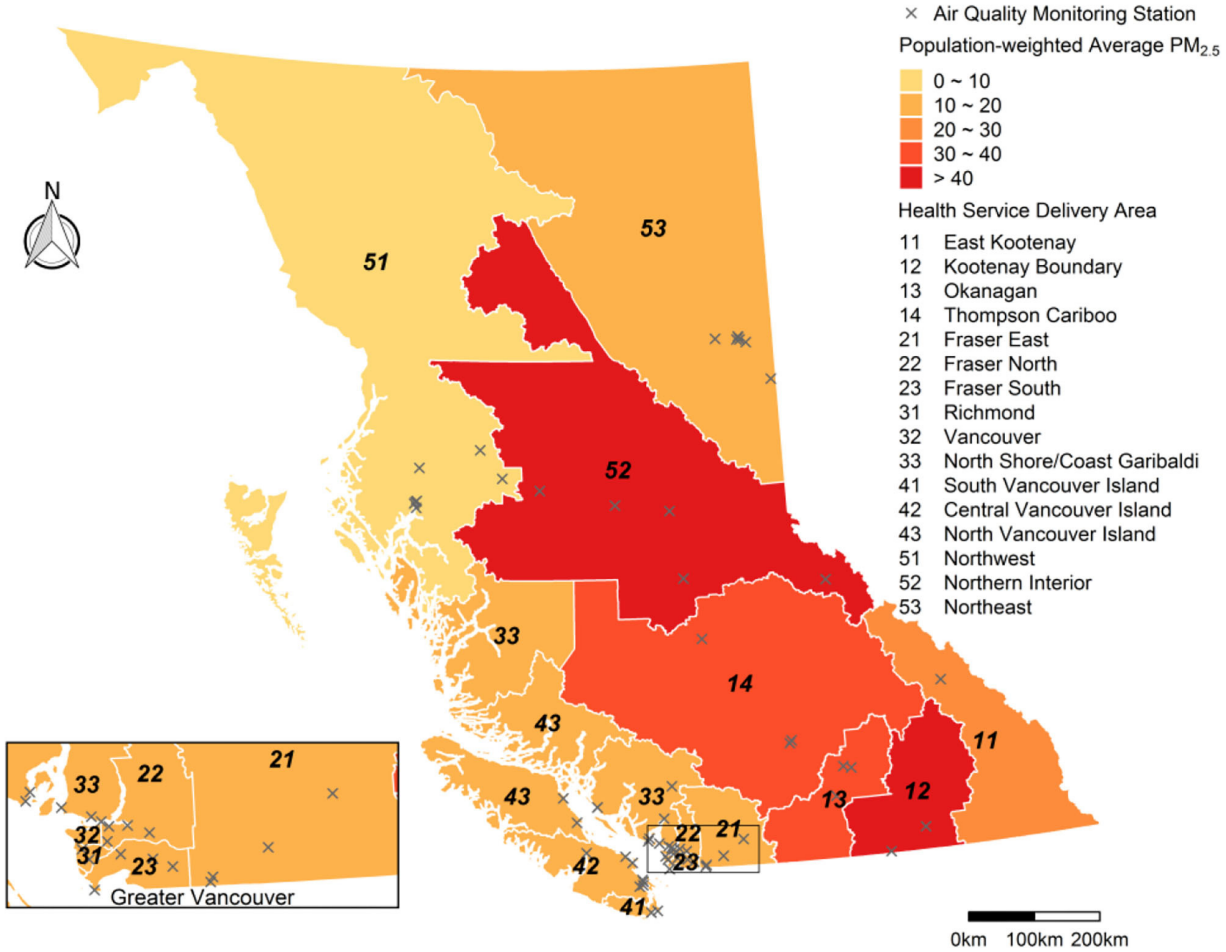
Map of British Columbia showing the 16 Health Service Delivery Areas (HSDAs) in the study area. The HSDAs are color-coded to show the population-weighted average of fine particulate matter (PM_2.5_) concentrations during the study period (15 July−15 September 2018) from the Optimized Statistical Smoke Exposure Model (OSSEM). Locations of the 62 regulatory air quality monitoring stations are also shown.

British Columbia is the westernmost province of Canada, with a total land area of 925,186 km^2^, and a population approaching 5.0 million people. Over half of this population resides in the coastal urban areas around greater Vancouver and greater Victoria, while most of the landmass is sparsely populated. Almost two-thirds of the province is heavily forested, with seasonal wildfire and smoke typically affecting the interior and northern regions. The province also has highly complex terrain bounded by the Coast Mountains in the west and the Rocky Mountains in the east, with the Interior Plateau between them (5). British Columbia is divided into 16 Health Service Delivery Areas (HSDAs) for the purposes of health administration, and these are used as the geographic units of analysis for BCAPS (Figure 2).

**Figure 3:**
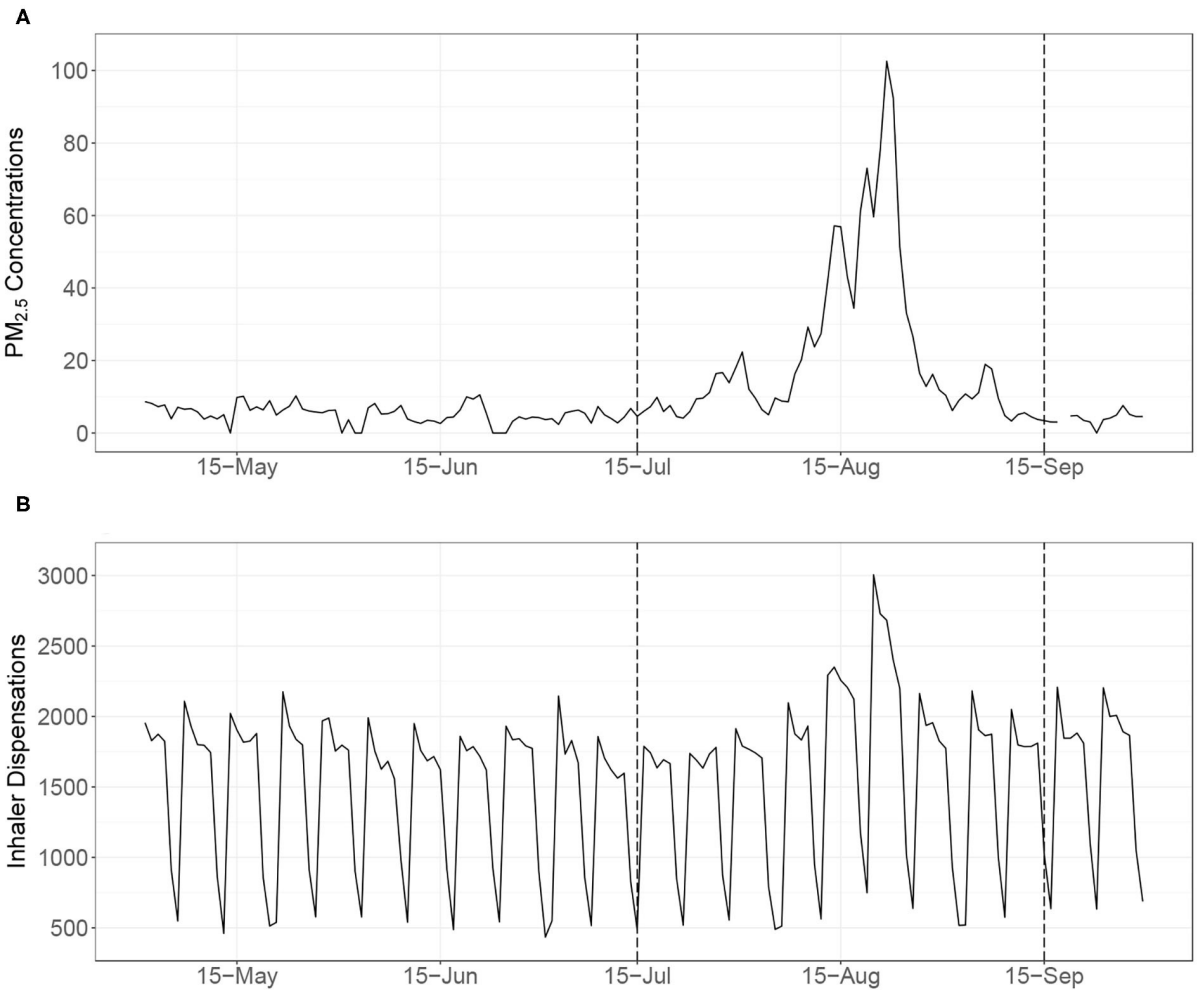
The upper time series **(A)** shows the daily population-weighted average of particulate matter (PM_2.5_) concentrations in British Columbia during the summer of 2018 from the Optimized Statistical Smoke Exposure Model (OSSEM). The lower time series **(B)** shows the daily province-wide counts of salbutamol sulfate (i.e., Ventolin®) inhaler dispensations, illustrating clear day-of-week differences. The 63-day study period for evaluation of the British Columbia Asthma Prediction System (BCAPS) is indicated by the dashed lines (15 July−15 September 2018).

From 1970 to 2016, the annual average (range) area burned by wildfire in British Columbia was 1,530 (120 – 3,690) km^2^, but the regime changed in 2017 when the area burned increased to 12,150 km^2^ and in 2018 when it increased again to 13,540 km^2^. The smoke impacts in 2017 were widespread, and BCAPS was launched midway through the season to help facilitate the public health response. The smoke impacts in 2018 were even more widespread and severe, providing an excellent opportunity to evaluate the performance of BCAPS under extreme conditions. The study covers a 63-day smoke period from July 15 to September 15, 2018 (Figure 3). The same period in the relatively mild wildfire season of 2016 is used to help contextualize some of the basic descriptive information about the air quality and health indicator impacts. Although the provincial population increased by 2.7% between 2016 and 2018, growth was concentrated in the greater Vancouver area (6).

### Module 1: Historic Daily PM_2.5_ Concentrations

Historic estimates of daily PM_2.5_ concentrations were taken from the Optimized Statistical Smoke Exposure Model (OSSEM), which was developed for real-time surveillance (3) and epidemiologic research (7) in British Columbia, as described in detail elsewhere (8). These estimates are generated for *day*_0_ (i.e., today) on a 5 × 5 km grid using the following variables:

- *PM*_2.5_: 24-hour average PM_2.5_ concentrations from the nearest of 62 regulatory air quality monitoring stations in the province as measured on *day*_−1_ (i.e., yesterday).
- *AOD*: Nearest aerosol optical depth (AOD) within 50 km, taken from the Moderate Resolution Imaging Spectroradiometer (MODIS) remote sensing instrument for *day*_−1_. The AOD is a unitless measure of aerosol in the atmospheric column, which can be correlated with PM_2.5_ at the surface (9).
- *FRP*: Average fire radiative power (FRP) within a 100 km radius for *day*_−1_, also taken from MODIS. The FRP indicates the rate of energy emitted from fires, which is proportional to their aerosol emissions (10).
- *HMS*: The hazard mapping system (HMS) includes smoke plume tracings generated from multiple remote sensing platforms by specialized analysts (11). We used these data to indicate binary smoke plume presence in a grid cell on *day*_−1_.
- *VI*: Venting index (VI) at the nearest of 29 stations on *day*_−1_. The VI ranges from 1 to 100 and is calculated by Environment and Climate Change Canada to indicate the ability of the atmosphere to disperse air pollution.

Data from the 2003 to 2012 wildfire seasons were used to train and test the implementation of OSSEM used by BCAPS in summer 2018. Briefly, all days during these seasons were classified as having low, moderate, or high smoke potential based on the provincial sum of FRP. On high smoke days, the linear regression model had an R^2^ value of 0.84, and a normalized root mean squared error (NRMSE) of 55%. When the overall model was evaluated with a leave-one-year-out cross-validation, the R^2^ value ranged from 0.41 – 0.83 (mean of 0.70) and the NRMSE ranged from 56.1 to 131.1% (mean of 84%). These results indicate that OSSEM is sensitive to the effects of a single wildfire season. During the 2003–2012 period, we also found that the highest PM_2.5_ concentration estimated by the model was 150 μg/m^3^, and only 12 of the 3,305 training observations were over this value. Thus, all of the training values over 150 μg/m^3^ were set to 150 μg/m^3^, and the model predictions were capped at 150 μg/m^3^ because it seemed to be a reasonable limit at the time (8). As such, the implementation of OSSEM used for the historic PM_2.5_ data in the 2018 implementation of BCAPS could not generate PM_2.5_ estimates over 150 μg/m^3^.

Daily OSSEM estimates were assigned to each HSDA for all dates in the wildfire seasons (April–October) of 2003–2017 using dissemination areas (DAs) from the 2016 national census, each of which has a population of 400 – 700 residents. We mapped the geographic center of each dissemination area (*N* = 7,617) in the study area, and then assigned PM_2.5_ based on the OSSEM value in the underlying grid cell. The population-weighted average for each HSDA was calculated by multiplying the PM_2.5_ estimates for all of its DAs against their populations, summing the results, and then dividing by the total HSDA population.

### Module 2: Historic Daily Counts of a Respiratory Health Indicator

Dispensations of salbutamol sulfate inhalers were used as the syndromic indicator of population respiratory health. Salbutamol sulfate (brand name Ventolin®) is a prescription drug used to treat the acute symptoms of asthma and chronic obstructive pulmonary disease (COPD), and previous research has shown increased inhaler dispensations during smoke events in British Columbia (12), though no information on their actual use is available. Other work has shown that the effect of PM_2.5_ on inhaler dispensations is similar to its effects on outpatient physician visits for asthma (7) and respiratory hospital admissions (13) in the province. Of these outcomes, dispensations of inhalers are most frequent, so they provide more power and sensitivity for surveillance modeling. The provincial government requires every prescription dispensation to be logged by the PharmaNet database (14), and aggregate counts for each HSDA are made available for public health surveillance (3). Historic inhaler dispensation counts were assigned for all dates in the wildfire seasons (April–October) of 2003–2017.

### Module 3: Predicted PM_2.5_ Concentrations for the Upcoming Days

The Canadian national FireWork smoke forecasting system uses current data to generate hourly forecasts of smoke-related PM_2.5_ at a spatial resolution of 10 × 10 km for the next 48 h. It is a complex framework that combines meteorological forecasts, fire locations, fuel consumption estimates, and smoke emissions estimates in a dispersion model to predict ground-level PM_2.5_ concentrations, among other variables (15). FireWork produces two smoke forecasts each day, and we used the 05:00 output for all analyses. The FireWork forecast for *day*_0_ (i.e., today) was derived by averaging the values for hours 1–24, and the forecast for *day*_+1_ (i.e., tomorrow) was derived by averaging the values for hours 25–48.

Previous work has demonstrated that wildfire smoke forecasts are valuable for both epidemiologic research (16) and public health surveillance (3). However, we have also demonstrated that the public health utility of FireWork was improved when the forecasts were blended with observation-based estimates from OSSEM, which generally serve to attenuate near-fire overestimates by FireWork (17). Blended PM_2.5_ forecasts for BCAPS were generated using a random forest model. This machine learning approach can provide accurate predictions while being robust to overfitting, accommodating non-linear relationships between the dependent and independent variables, and accounting for complex interactions between the independent variables (18).

The dependent variable for the *day*_0_ (i.e., today) BCAPS forecast was the observed 24-h average PM_2.5_ concentration at the 62 regulatory PM_2.5_ monitoring stations across the province (Figure 2), with very high concentrations truncated to 150 μg/m^3^ as done for OSSEM to ensure internal consistency. Six independent variables at the same 62 locations were offered to the random forest model: the *day*_0_ FireWork forecast and all five of the *day*_−1_ variables used in OSSEM (PM_2.5_, AOD, FRP, HMS, and VI). We fitted the model with 1,000 regression trees and a subset of three predictive variables sampled for each tree, using the *randomForest* package in R (19). Each tree was trained with a random subset of the data and predictions were made for the remaining observations, otherwise known as the “out-of-bag” data. Because each observation was out-of-bag multiple times for the 1,000 trees, it had multiple predicted values for the observed PM_2.5_ on *day*_0_. The averages of the out-of-bag predictions for each observation were used to calculate the root mean square error (RMSE, Equation 1) and the pseudo R-squared value (percentage of variance explained) for the model.

(1) $$\begin{array}{r} \text{RMSE}=\sqrt{\frac{\overset{n}{\underset{i\;=\;1}{\sum }}{\left(P_{i}-O_{i}\right)}^{2}}{n}} \end{array}$$

Where *P*_*i*_ is the average of the out-of-bag predictions for the observed *O*_*i*_ value.

The RMSE for the model used to generate the 2018 BCAPS *day*_0_ blended PM_2.5_ forecast was 4.7 μg/m^3^ and the pseudo *R*^2^ value was 0.85. The *day*_+1_ (i.e., tomorrow) blended PM_2.5_ forecast for BCAPS was generated by averaging the *day*_0_ blended forecast with the *day*_+1_ FireWork forecast. Once the calculations were complete, the maximum value within each HSDA was extracted for the health forecasts. The maximum value was chosen over the population-weighted average so that the health forecasts reflected the worst case scenario.

### Module 4: Model to Forecast Counts of the Respiratory Health Indicator for the Upcoming Days

Another random forest model was used to predict inhaler dispensation counts for *day*_0_ (i.e., today) and *day*_+1_ (i.e., tomorrow) in each HSDA. For the sake of efficiency, the training data included all dates with PM_2.5_ concentration estimates over 15 μg/m^3^, and a random sample of dates with lower PM_2.5_ concentrations. The base model was trained on the observed counts using the following independent variables: (1) the *day*_0_ (i.e., today) population-weighted PM_2.5_ observation from OSSEM; (2) the *day*_−1_ (i.e., yesterday) population-weighted PM_2.5_ observation from OSSEM; (3) the average dispensation count observed on the same day-of-week for the past 12 weeks, accounting for holidays (i.e., long-term trend); (4) the average dispensation count observed on the same day-of-week for the past 4 weeks, accounting for holidays (i.e., short-term trend); and (5) the week of year. The day-of-week and holiday indicators are required because many pharmacies are closed on Saturdays, Sundays, and holidays, which leads to strong weekly patterns in the data (Figure 3). The model RMSE was 17.6 dispensations, and the pseudo *R*^2^ value was 0.94. Once the model was trained, it was used to make health forecasts for *day*_0_ (i.e., today) by replacing variable (1) with the blended *day*_0_ PM_2.5_ forecast. The health forecasts for *day*_+1_ (i.e., tomorrow) were made by replacing variable (1) with the blended *day*_+1_ PM_2.5_ forecast and variable (2) with the blended *day*_0_ PM_2.5_ forecast.

### Module 5: Report to Visualize Inputs and Outputs

**Figure 4:**
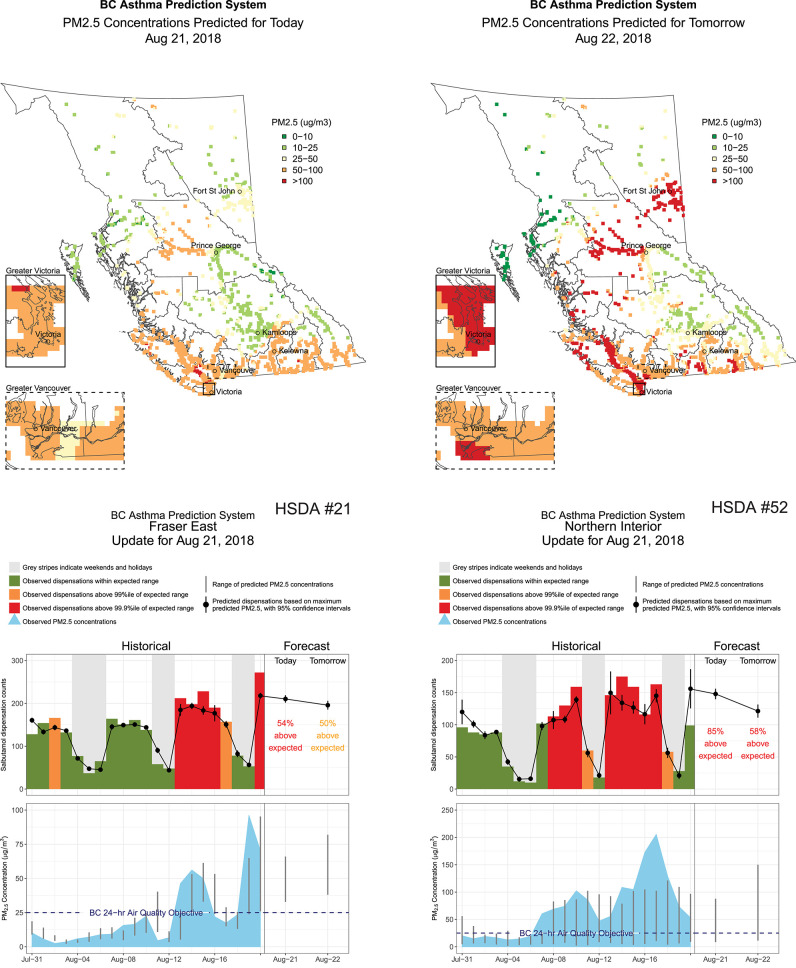
Four pages extracted from the British Columbia Asthma Prediction System (BCAPS) report disseminated to public health users on August 21, 2018. The maps show the blended forecast for fine particulate matter (PM_2.5_) concentrations on August 21, 2018 (i.e., today) and August 22, 2018 (i.e., tomorrow) across all populated 5 × 5 km grid cells in the province. The range of PM_2.5_ forecasts within the two Health Service Delivery Areas (HSDAs) is shown as vertical gray lines on the far right-hand side of the lower charts. The top of the range was used to make the health forecasts on the far right-hand side of the upper charts. The historical information on the left-hand side of the lower and upper charts shows how BCAPS forecasts have compared with observations over the previous 3 weeks, where the observed PM_2.5_ concentrations are taken from the provincial air quality monitoring network.

All input data and model output were visualized in a daily report for each HSDA showing the historic record of observed PM_2.5_ and inhaler dispensations for the past 21-day period on the left-hand side, and the forecasted PM_2.5_ and inhaler dispensations for the upcoming 2-day period on the right-hand side. As the wildfire season progresses, this design allows users to evaluate how the BCAPS framework has performed (Figure 4).

### Evaluation of BCAPS Performance During the 2018 Wildfire Season

A tool such as BCAPS is only useful if its users can have confidence in its predictions. Therefore, the utility of BCAPS depends primarily on the accuracy of the health forecasts and secondarily on the accuracy of the PM_2.5_ forecasts, which comprise two of the five variables in the health forecasting model. To examine the accuracy of these predictions we calculated the RMSE and index of agreement (IOA, Equation 2) for the *day*_0_ (i.e., today) and *day*_+1_ (tomorrow) inhaler and PM_2.5_ forecasts over the 63-day study period in each HSDA, as well as the population-weighted values. The IOA ranges from 0 to 1, where 0 indicates no agreement and 1 indicates perfect agreement. For the inhaler forecasts we expressed the RMSE as a rate per 10 000 population in each HSDA. We also calculated the number of days for which the inhaler forecasts were within 20% of the inhaler observations, because this may be a more intuitive measure for applied public health users.

(2) $$\begin{array}{r} \text{IOA}=1-\left[\frac{\overset{n}{\underset{i\;=\;1}{\sum }}{\left(P_{i}-O_{i}\right)}^{2}}{\overset{n}{\underset{i\;=\;1}{\sum }}{\left(\left|P_{i}-\bar{O}|+|O_{i}-\bar{O}\right|\right)}^{2}}\right] \end{array}$$

The wildfire season of 2018 was unprecedented with respect to its air quality impacts. Because OSSEM and the blended PM_2.5_ forecasts were both effectively capped at 150 μg/m^3^, OSSEM could not provide a fair assessment of any under-prediction by the PM_2.5_ forecasts. As such, the PM_2.5_ forecasts were evaluated against the 24-h average PM_2.5_ measurements taken at 62 regulatory air quality monitoring stations in the province (Figure 2). The population-weighted average for all stations within each HSDA was compared with the maximum forecast value for the HSDA, which is consistent with the data presented in the BCAPS reports (Figure 4).

## Results

### Observed PM_2.5_ Concentrations and Inhaler Counts

The mean (range) of daily province-wide population-weighted PM_2.5_ concentrations from OSSEM was 22.0 (3.3–103) μg/m^3^ over the 63-day study period (Table 1, Figure 3). As a comparison, the same values for the relatively mild wildfire season of 2016 were 4.2 (2.0–8.0) μg/m^3^. The least smoke-impacted HSDA in 2018 was the Northwest (#51), with a mean of 10.5 (3.1–71.8) μg/m^3^ and the most smoke-impacted HSDAs was Kootenay Boundary (#12) with a mean of 47.1 (4.6–150) μg/m^3^ (Table 1). The maximum 24-h average concentration measured by the 62 regulatory air quality monitoring stations was 883 μg/m^3^. The 99th percentile was 215 μg/m^3^ and the 98th percentile was 148 μg/m^3^, meaning that approximately 2% of all surface PM_2.5_ measurements during the study period were over the 150 μg/m^3^ cap on the OSSEM PM_2.5_ estimates and the blended PM_2.5_ forecasts.

**Table 1:** Summary information for the 16 Health Service Delivery Areas (HSDAs) in British Columbia during the 63-day study period (15 July−15 Sep 2018) and a similar period during a relatively mild wildfire season (July 15–September 15, 2016).

**HSDA**	**HSDA name**	**2018 population**	**2018 PM_**2.5**_ from OSSEM (μg/m^**3**^)**	**2016 PM_**2.5**_ from OSSEM (μg/m^**3**^)**	**Total inhaler dispensations in 2018**	**Percent increase over 2016 inhaler dispensations**
11	East Kootenay	84,594	45.5	4.4	2,245	31%
12	Kootenay Boundary	82,620	47.1	4.3	2,170	38%
13	Okanagan	387,135	33.4	4.8	8,988	49%
14	Thompson Cariboo	234,874	34.5	4.9	7,475	39%
21	Fraser East	319,023	19.9	4.7	8,087	30%
22	Fraser North	685,094	18.5	4.6	10,895	20%
23	Fraser South	856,681	18.8	4.6	15,256	29%
31	Richmond	216,300	18.7	4.7	2,255	27%
32	Vancouver	692,228	18.5	5.3	9,324	20%
33	North Shore/Garibaldi	302,363	18.0	4.6	5,116	24%
41	South Vancouver Island	413,406	18.0	2.8	7,190	29%
42	Central Vancouver Island	291,209	17.4	3.0	7,855	29%
43	North Vancouver Island	131,256	15.5	2.4	3,294	27%
51	Northwest	75,104	10.5	3.8	1,558	7%
52	Northern Interior	148,845	44.8	5.2	4,962	50%
53	Northeast	70,955	20.4	4.7	1,743	22%
All	All (population-weighted)	4,991,687	22.0	4.2	99,439	30%

*Population-weighted estimates of fine particulate matter (PM_2.5_) concentrations were taken from the Optimized Statistical Smoke Exposure Model (OSSEM). Shading indicates HSDAs in the same health region. The population increase between 2016 and 2018 was 2.7%, concentrated in HSDAs #22, #23, #31, and #32*.

The province-wide sum of inhaler dispensations during the 63-day study period was 99,406, a 30% increase over the 76,583 inhalers dispensed during the same period of the milder 2016 wildfire season (Table 1). The HSDA with the smallest increase over 2016 values was the Northwest (#51) at 7%, while the HSDA with the largest increase was the adjoining Northern Interior (#52) at 50%. Given that two forecasts of inhaler dispensations were made on each day (i.e., one for today and one for tomorrow), the total number of predictions for each HSDA was 126. Of these, an average of 71% were within 20% of inhaler dispensations that were actually observed, though this ranged from 50% in the East Kootenay (#11) to 91% in Fraser South (#23), which is the most populous HSDA. Overall, there was a positive correlation (Pearson *R* = 0.73) between HSDA population and the percent of inhaler dispensation forecasts within 20% of the observed value.

### Performance of the Blended PM_2.5_ Forecasts

**Figure 5:**
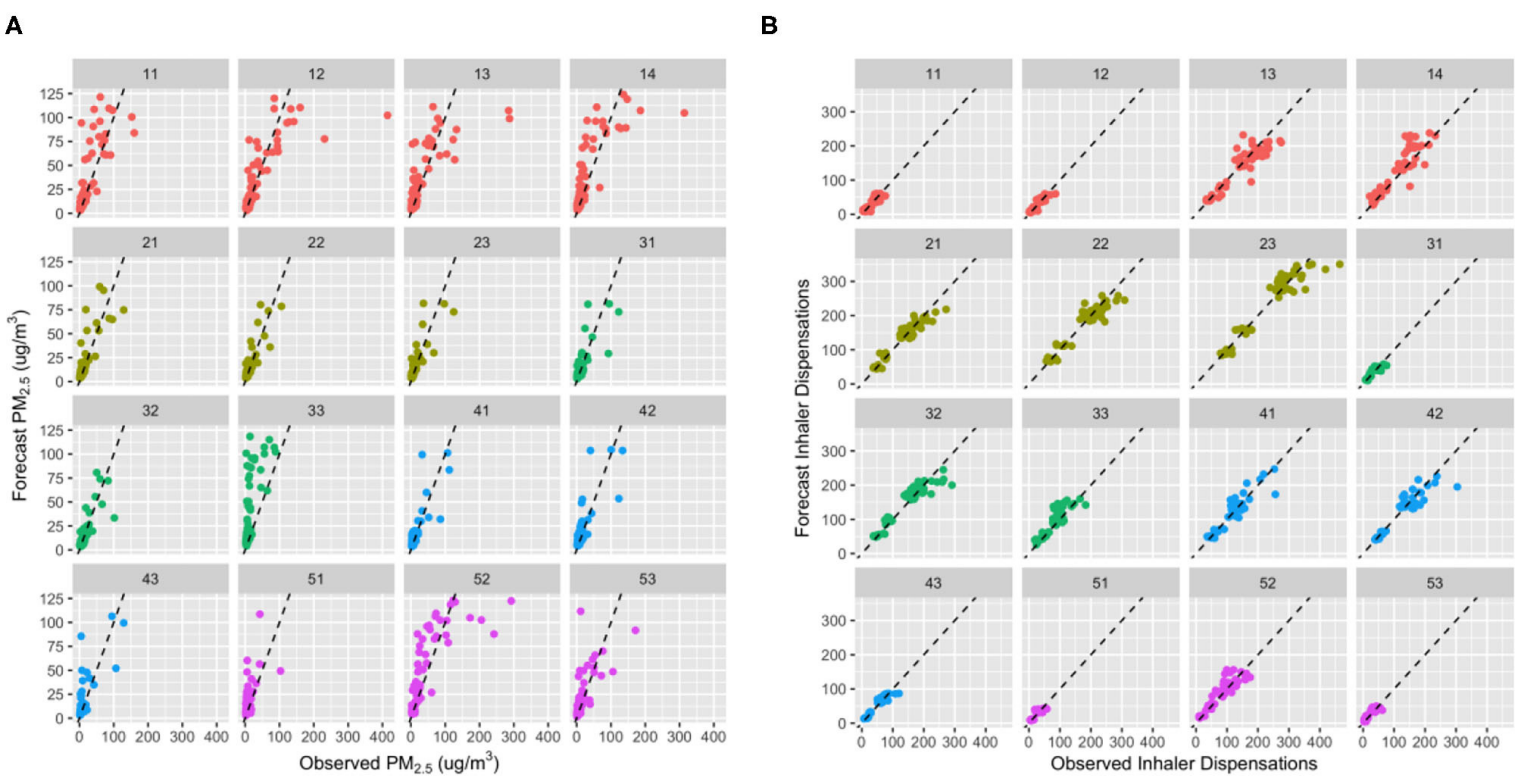
Scatter plots showing the *day*_0_ (i.e., today) agreement between observed and forecast values for **(A)** fine particulate matter (PM_2.5_) and **(B)** inhaler dispensation counts for each of the 16 Health Service Delivery Areas (HSDAs). The points are color coded to group HSDAs by health region, and each plot shows a dashed 1:1 line for reference. For **(B)**, differences in population sizes between the HSDAs are made evident by differences in the range of inhaler dispensations counts. Bi- or tri-modal distributions in the larger HSDAs are due to differences between weekdays and weekends, when many pharmacies are closed.

Performance of the blended PM_2.5_ forecasts was moderate overall, but markedly different between HSDAs (Figure 5). For the interior HSDAs most affected by severe smoke (#12, #13, #14, and #52), the blended PM_2.5_ forecasts underpredicted the highest exposures, which was not surprising given the 150 μg/m^3^ cap on the input data for the random forest model. On the other hand, the blended PM_2.5_ forecast also systematically overpredicted exposures in some HSDAs (#11, #14, #33, #51, and #52) when the forecasts were compared with measurements from the available regulatory air quality monitoring stations. Such apparent overprediction is likely due to the fact that the maximum forecast value for the entire HSDA is being compared with measurements taken at a limited number of locations that may not reflect smoke impacts due to positioning and topography (Figure 2).

**Figure 6:**
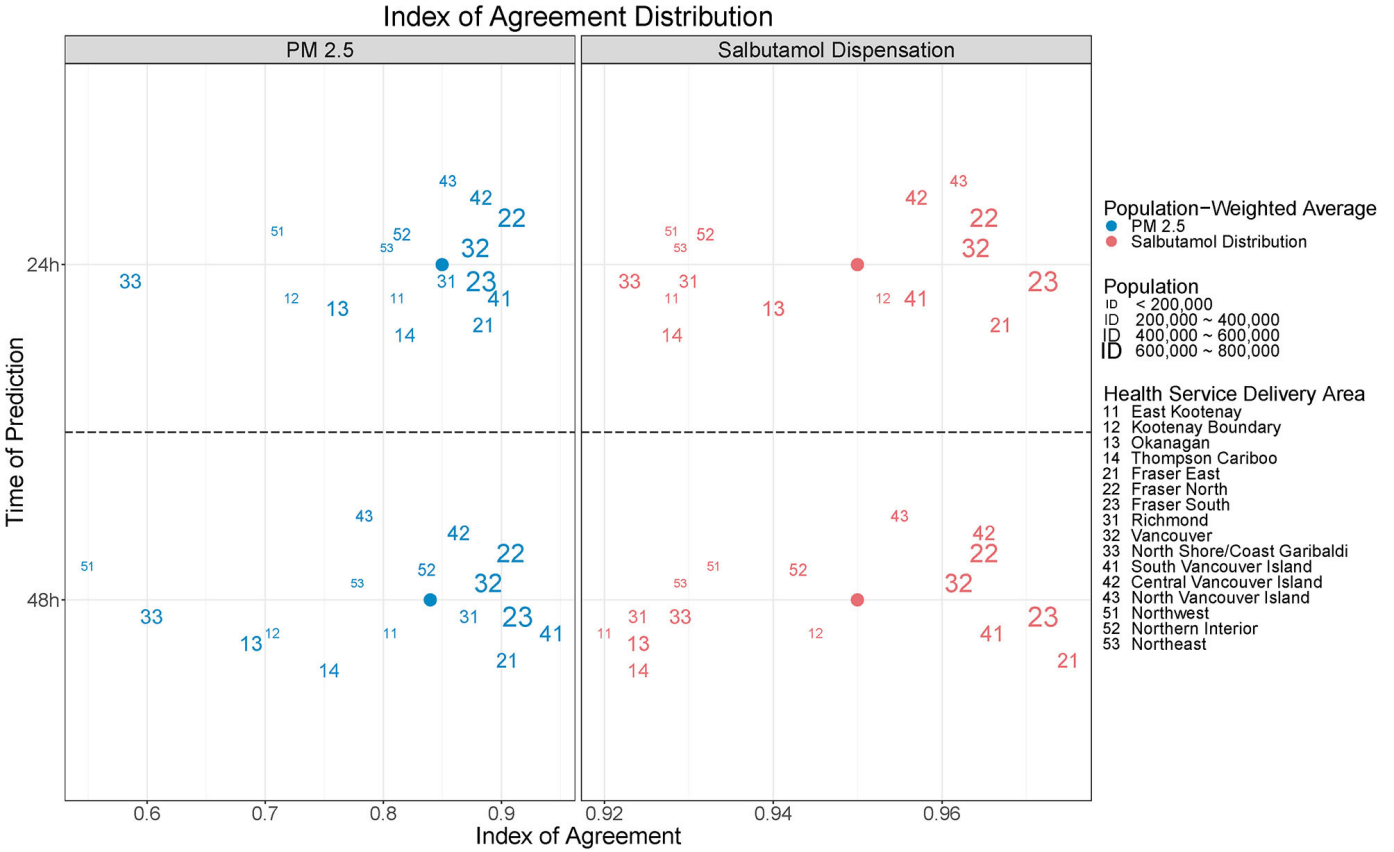
The index of agreement (IOA) was used to compare daily blended forecasts for fine particulate matter (PM_2.5_) concentrations and salbutamol sulfate (i.e., Ventolin®) inhaler dispensations. Values are shown for the forecasts made for today (*day*_0_, top) and tomorrow (*day*_+1_, bottom).

There was minimal difference in performance of the blended PM_2.5_ forecasts between *day*_0_ (i.e., today) and *day*_+1_ (i.e., tomorrow). The population-weighted mean (range) IOA values were 0.85 (0.58–0.92) and 0.85 (0.55–0.94), respectively (Figure 6). Agreement was markedly lower in those HSDAs where the blended PM_2.5_ forecasts systematically overpredicted the measured concentrations (#12, #13, #33, and #51) whereas agreement was more moderate in those HSDAs where the measurements were underpredicted. There was a moderate positive correlation between the IOA and HSDA population (Pearson *R* = 0.50). The population-weighted mean (range) RMSE for all 126 blended PM_2.5_ forecasts in each HSDA was 20.5 (10.9–51.8) μg/m^3^, which decreased to 17.9 (10.9–30.0) μg/m^3^ when observations over 150 μg/m^3^ were omitted. The RMSE had a moderate negative correlation with HSDA population (Pearson *R* = −0.51), indicating smaller errors in HSDAs with larger populations.

### Performance of the Inhaler Count Forecasts

Performance of the inhaler dispensation forecasts was strong overall, and more consistent across the HSDAs than the performance of the blended PM_2.5_ forecasts (Figure 5). Although some systematic over- and underprediction was apparent in the HSDAs where PM_2.5_ was over- and underpredicted, the magnitude of the PM_2.5_ differences was attenuated by the inhaler dispensation forecasts (Figure 3 shows a clear example in HSDA #52). This is reasonable, given that the PM_2.5_ forecasts contribute only two of the five variables in the random forest health forecasting model.

**Figure 7:**
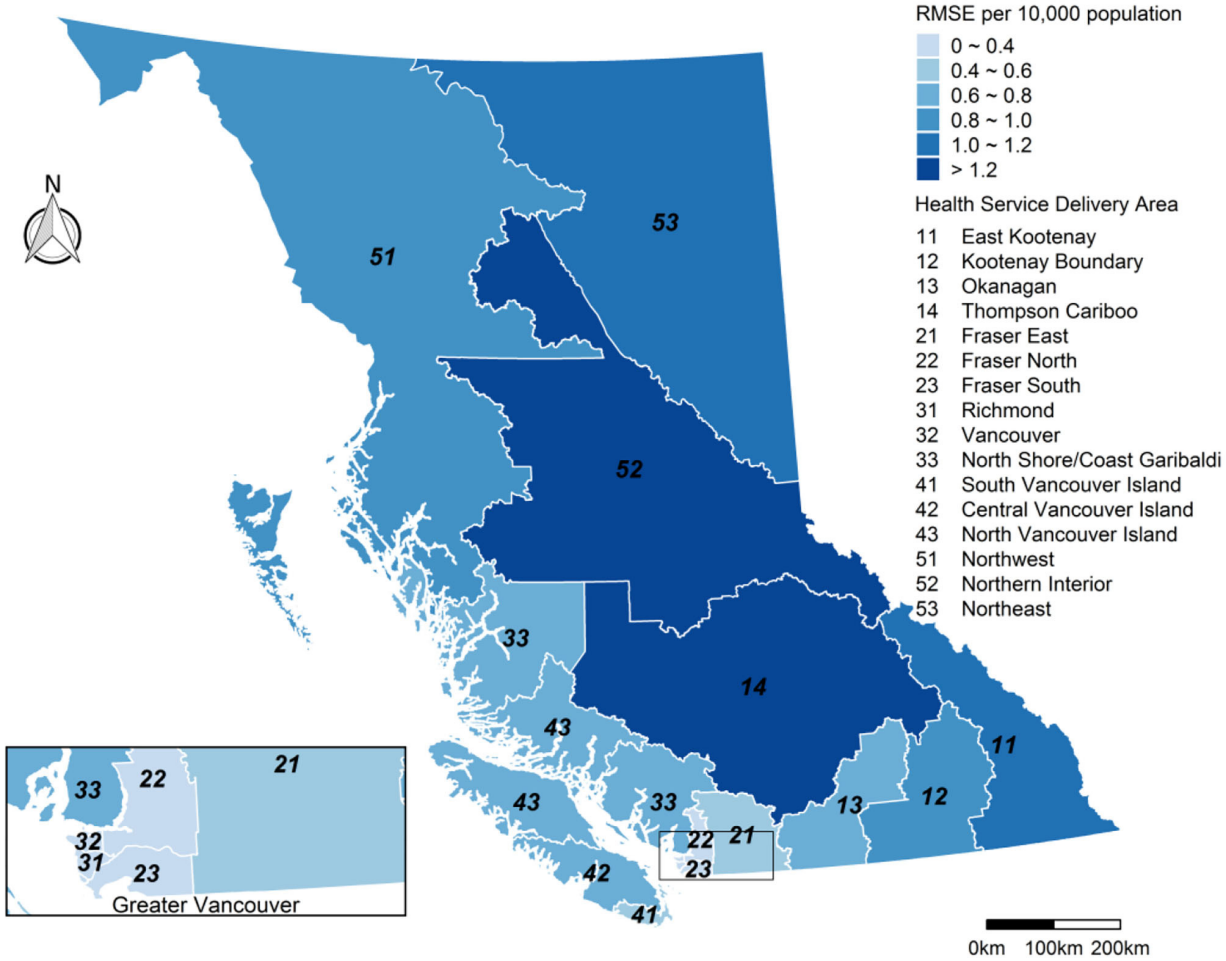
The root mean squared error (RMSE) for the inhaler dispensation forecasts per 10 000 population in each Health Service Delivery Area (HSDA) during the study period (15 July−15 September 2018). Summary includes all forecasts for *day*_0_ (i.e., today) and *day*_+1_ (i.e., tomorrow).

The strength of the overall inhaler dispensation forecasts was reflected in the population-weighted mean (range) IOA, which was 0.95 (0.92–0.98) for both the *day*_0_ and *day*_+1_ forecasts (Figure 6). Once again, there was a moderate positive correlation between IOA and HSDA population (Pearson R = 0.64) when considering all 126 forecasts. The population-weighted mean (range) RMSE per 10,000 persons was 0.56 (0.33–1.37) inhaler dispensations. The largest values were in the Northern Interior (#52) and the adjacent Thomson Cariboo (#14), both of which are located in the provincial interior where the smoke impacts were the most extreme (Figure 7). Indeed, the correlation between the inhaler dispensation RMSE per 10,000 and the blended PM_2.5_ forecast RMSE was quite high (Pearson *R* = 0.70).

## Discussion

The operational performance of the modular BCAPS framework has been evaluated during the extreme wildfire season of 2018 in British Columbia, Canada. Comparison between observed PM_2.5_ measurements and the blended PM_2.5_ forecasts found moderate agreement, with systematic underprediction of the very high concentrations observed in the most smoke-impacted areas. Agreement was stronger for the larger populations in areas with more moderate smoke impacts. Comparison between the observed and forecast inhaler dispensations found strong agreement, despite moderate performance of the PM_2.5_ forecasts. The majority of forecasts were within 20% of the observed value with an overall tendency toward underprediction despite use of the maximum PM_2.5_ for each HSDA in the forecasting model. Although we assessed the forecasts for *day*_0_ (i.e., today) and *day*_+1_ (i.e., tomorrow) separately, we found little difference in performance between them (Figure 6).

Forecasting the air quality impacts of wildfire smoke over the coming days is a challenging problem, but also an active area of research where rapid advancements are being made. For example, a new version of FireWork was launched in 2019, which shows performance improvements over the version available in 2018 (20). Regardless, the quality of smoke forecasts will always rely on the quality of the model inputs, each of which has its own large uncertainties in the complex terrain of British Columbia. Key inputs for FireWork include fire locations and growth, fire emissions, atmospheric injection height of the emissions, and weather forecasts (21). Despite the challenges and uncertainties, smoke forecasts have the potential to be a very valuable resource for public health authorities seeking to understand the potential impacts of upcoming wildfire smoke exposures.

Improving smoke forecasts for the purposes of public health surveillance is an active area of research in British Columbia and elsewhere (17, 22, 23). The blended PM_2.5_ forecasts used in BCAPS during the summer of 2018 reflect previous work demonstrating that FireWork output was more strongly associated with health outcomes in British Columbia when blended with observation-based estimates from OSSEM, which served to attenuate near-fire overestimates (17). Even so, more sophisticated approaches could be run within the BCAPS framework to improve the smoke forecasts using the historic PM_2.5_ observations. Specifically, *Module 3* could be updated to model the historic relationship between PM_2.5_ observations and FireWork PM_2.5_ forecasts, and the results could be applied directly to the FireWork forecasts before passing them to *Module 4*. This would alleviate the need for the AOD, FRP, HMS, and VI data to be available in near-real-time when BCAPS is run each morning.

The most challenging aspect of this assessment was choosing the PM_2.5_ observations against which to compare the blended PM_2.5_ forecasts. Although PM_2.5_ estimates from OSSEM are generated on the same 5 × 5 km grid as the blended PM_2.5_ forecasts, both OSSEM and the forecasts were effectively capped at 150 μg/m^3^, so OSSEM could not be used to illustrate significant underprediction by the blended PM_2.5_ forecasts when present. Instead, we chose to compare the blended PM_2.5_ forecasts with the average PM_2.5_ concentrations measured by all regulatory air quality monitoring stations in each HSDA, which was consistent with reports sent to BCAPS users (Figure 4). However, this leads to spatial discrepancies in large HSDAs where the locations of the regulatory stations may not match areas of greatest smoke impact. Overall, the evaluation clearly demonstrated that the 150 μg/m^3^ cap was inappropriate in the changing wildfire regime of British Columbia, and that OSSEM and the blended PM_2.5_ forecasts required updating. Similarly, the historic training data for health forecasting model did not include any PM_2.5_ concentrations approaching those observed or forecasted during the case study period. As such, there was no information about the previous response of the target population to such high exposures, which may have attenuated the forecasts for the inhaler dispensations.

For summer 2019 we rebuilt the 24-h version of OSSEM using data from 200 to 2018 and machine learning methods similar to those for our 1-h model developed for research purposes (24). In summer 2019 we also started using raw FireWork estimates in *Module 3*, after significant improvements to the forecasting framework (20). The health forecasting model was also retrained, and the entire system was moved to an interactive online platform (https://maps.bccdc.ca/bcaps/). However, the 2019 updates have not been retrospectively applied to these analyses because our objective was to evaluate the operational version of BCAPS in 2018. Perhaps the most important conclusion of this study is that BCAPS need to be constantly evaluated and adapted as environmental conditions, population susceptibilities, and smoke forecasting systems change. Ideally, the models that underlie such systems should be re-trained after every severe wildfire season. A very advanced system could be re-trained daily to adapt to new data, though it would be computationally expensive.

The statistical approach we used for the health forecasting was a machine learning random forest model that predicts inhaler dispensations based on week-of-year, short- and long-term day-of-week temporal trends, holidays, and PM_2.5_ forecasts. Although performance of this model was strong on the training data and in BCAPS during the summer of 2018, machine learning is a black box approach to forecasting despite its growing use in air pollution epidemiology (25). Unlike a conventional linear regression model, it is difficult to interpret the role that any one variable plays in a random forest model which makes it difficult to explain why the health forecasts were relatively robust to large PM_2.5_ underpredictions in the most smoke-impacted HSDAs. One possible explanation is that BCAPS can easily forecast the strong day-of-week effect in the data, such that the skill of the model would already be very high in the absence of smoke-impacted PM_2.5_ concentrations. This could be tested by replacing the PM_2.5_ forecasts we used with more typical background values, or with estimates from other forecasting methods. Another possible explanation would be a non-linear relationship between PM_2.5_ and inhaler dispensations at high concentrations, which the random forest approach is designed to accommodate (18). Such log-linear relationships are often evident in air pollution epidemiology (26), and this issue requires more attention in the literature on wildfire smoke as wildfire seasons worsen under climate change.

## Public Health Implications

Wildfire smoke is an increasingly important public health challenge (27), and responders need useful tools to facilitate evidence-based decision-making during smoke events. Operational smoke forecasts provide valuable information about upcoming conditions, but these data are not readily accessible or inherently meaningful to most public health users (4). The BCAPS framework routinely and systematically integrates smoke forecasts with data from other sources to predict how specific populations will respond to impending smoke exposures based on their prior response. The framework is flexible and scalable, meaning that it can be used with a wide range of input data, predictive models, and visualization methods, and that it can be applied to any population. The framework is also adaptable, meaning that insight from annual evaluation can be used to improve BCAPS for future wildfire seasons.

Overall, this *quantitative* evaluation found that daily reports from the BCAPS framework provided timely and reasonable insight into the population health impacts of forecasted smoke exposures during the extreme 2018 wildfire season, though more work is necessary to improve the PM_2.5_ models in future. However, we have not provided a *qualitative* evaluation of how the BCAPS reports were used by their recipients, which is another important consideration for any such surveillance product. Based on recent work by colleagues in British Columbia, we know that BCAPS users have identified the need for more publicly accessible monitoring systems, especially in rural and remote communities (28). To address this need, the newest version of BCAPS has been made available in a dynamic online environment that is optimized for mobile platforms (https://maps.bccdc.ca/bcaps/). The evidence generated by this evaluation has already led to improved performance of the current BCAPS implementation.

## Data Availability Statement

The datasets for this article are not publicly available because they include personal health records. Requests to access the datasets should be directed to Sarah Henderson – sarah.henderson@bccdc.ca.

## Author Contributions

SH conceptualized the work and led the writing of the manuscript. KTM, KEM, YD, and JY developed the surveillance system, conducted the analyses, and reviewed the manuscript. GS and DB provided guidance on the development of the surveillance system, provided supervision for KTM, and reviewed the manuscript. All authors contributed to the article and approved the submitted version.

## Conflict of Interest

The authors declare that the research was conducted in the absence of any commercial or financial relationships that could be construed as a potential conflict of interest.
